# Modelling health systems barriers to successful malaria management

**DOI:** 10.1186/1475-2875-11-S1-P126

**Published:** 2012-10-15

**Authors:** Bhargavi Rao, David Schellenberg, Azra Ghani

**Affiliations:** 1 Department of Infectious Disease Epidemiology, Imperial College London, UK; 2 Department of Disease Control, Faculty of Infectious Diseases, London School of Hygiene and Tropical Medicine, UK

## Background

The success of national malaria control strategies is increasingly recognised to be limited by the capacity of the health system to deliver interventions at the required levels of coverage and quality [1]. It is critical to better understand how to deliver a proven intervention, such as Artemisinin Combination therapies (ACTs), most effectively through an existing system, and where the barriers are to achieving its predicted potential. Few models address the delivery of case management: the Piot model [2] or the “community effectiveness framework” [3] has been used to describe how a cascade of interacting health-systems barriers may sequentially reduce the effectiveness of treatment interventions. [4,5] However the end-estimate of correct treatment is lower than estimates from health facility surveys. [1,4-12] We investigated whether an alternative decision-tree approach may more closely estimate rates of appropriate treatment for malaria and also non-malarial febrile illness (NMFI).

## Methods

We systematically reviewed the sequential process of and barriers to, malaria case management through primary health care in sub-Saharan Africa. We thus developed community effectiveness and decision tree models to case management in the public sector. Articles published since the rollout of ACTs were used to obtain a range of estimates for malaria management steps. Parameters were sampled from between these ranges of estimates and used to inform the two models. We then explored scenarios of lifting different case management barriers.

## Results

The decision analysis model more accurately reflected reported levels of appropriate management of fever (malarial and non-malarial) in the public sector (>60% attendees), compared with a systems effectiveness approach (<2%). Scenarios of perfect case management steps all improved correct fever management using both models, except in the case of perfect ACT stock; a decision tree approach predicted a 12% reduction in correct management of all cases compared with a 63% increase with the community effectiveness approach.

Using the decision-tree model, increases in availability and rapid diagnostic tests (RDTs) improved overall management of fever upto 65% of all attendees, and reduced overtreatment of NMFI with unnecessary antimalarials by over 35%, but did not substantially improve appropriate treatment rates of malaria cases. The greatest impact on the proportion of malaria cases correctly treated resulted from improving ACT stock (over 65% increase from baseline), even without increased RDT use. Under conditions of perfect availability and use of RDTs, test adherence and drug availability appropriate treatment rates rose to over 85%.

## Conclusion

The relationship between improving delivery through health systems and resulting impact on health outcomes of infectious diseases is not straightforward, however simple decision analysis models can provide insight into which aspects of delivering care are most likely to impact on care quality and treatment effectiveness. Unlike the community-effectiveness framework, the decision-tree approach accounts for clinical (non-RDT guided) treatment of malaria, under-diagnosis of malaria and unnecessary antimalarial use in NMFI cases, which are all of policy interest impacting on health outcomes and ongoing transmission. Further work into the amenability of health systems to change is required to explore the most cost-effective targets in expanding the delivery of antimalarials.
